# Prognostic Value of ^**18**^F-FDG PET-CT in Nasopharyngeal Carcinoma: Is Dynamic Scanning Helpful?

**DOI:** 10.1155/2015/582614

**Published:** 2015-04-30

**Authors:** Bingsheng Huang, Ching-Yee Oliver Wong, Vincent Lai, Dora Lai-Wan Kwong, Pek-Lan Khong

**Affiliations:** ^1^Department of Diagnostic Radiology, The University of Hong Kong, Hong Kong; ^2^Department of Diagnostic Radiology and Molecular Imaging, Oakland University William Beaumont School of Medicine, Royal Oak, MI 48073, USA; ^3^Department of Clinical Oncology, Queen Mary Hospital, Hong Kong

## Abstract

*Objectives*. To evaluate the differences in prognostic values of static and dynamic PET-CT in nasopharyngeal carcinoma (NPC). *Material and Methods*. Forty-five patients who had static scan were recruited. Sixteen had dynamic scan. The primary lesions were delineated from standardized uptake value (SUV) maps from static scan and *K*_*i*_ maps from dynamic scan. The average follow-up lasted for 34 months. The patients who died or those with recurrence/residual disease were considered “poor outcome”; otherwise they were considered “good outcome.” Fisher's exact test and ROC analysis were used to evaluate the prognostic value of various factors. *Results*. Tumor volume thresholded by 40% of maximal SUV (VOL_SUV40_) significantly predicted treatment outcome (*p* = 0.024) in the whole cohort. In 16 patients with dynamic scan, all parameters by dynamic scan were insignificant in predicting the outcome. The combination of maximal SUV, maximal *K*_*i*_, VOL_SUV40_, and VOL_*K*_*i*_37_ (the tumor volume thresholded by 37% maximal *K*_*i*_) achieved the highest predicting accuracy for treatment outcome with sensitivity, specificity, and accuracy of 100% in these 16 patients; however this improvement compared to VOL_SUV40_ was insignificant. *Conclusion*. Tumor volume from static scan is useful in NPC prognosis. However, the role of dynamic scanning was not justified in this small cohort.

## 1. Introduction

Nasopharyngeal carcinoma (NPC) is an aggressive head and neck cancer that is common in Southern China and South East Asia. Positron emission tomography-computed tomography (PET-CT) using ^18^F-fluoro-2-deoxy-D-glucose (FDG), namely, FDG PET-CT, provides combined anatomic and metabolic information in one examination. Studies have shown that PET-CT has the potential value in the early treatment and posttreatment assessment by improving the differentiation between residual/recurrent disease and postchemoradiation fibrosis [1, 2] and may serve as prognostic indicators especially in patients with locoregionally advanced disease [3]. Standardized uptake value (SUV) and the metabolic tumor volume have been demonstrated to be useful for prognostication in NPC patients in some studies but these findings have not been consistent [4–9]. This may be due to limitations of SUV-based parameters as follows; SUV is calculated by dividing the FDG concentration of a voxel (or a region) at a single time point by the administered FDG activity normalized to a measure of distribution volume such as body weight, mass, or volume. It can be affected by various factors such as the amount of dose administered, condition of the body such as excretion rate of the tracer and body fat content, and the uptake time after tracer administration [10]. Also, metabolic tumor volume, usually acquired by using a SUV value as the threshold, and the delineation of tumor margins based on SUV maps are influenced by the factors that affect the accuracy of SUV measurement [11, 12]. Moreover, as SUV is a relative measurement, the parameters derived from it are specific to a machine and cannot be applied to different machines.

To mitigate the shortcomings of SUV, especially to address the single time-point and static nature of this method, absolute quantitative analytic methods using dynamic PET-CT scan have been applied for studying the pattern of FDG metabolism in tissue [13–15]. These methodologies, first developed by Phelps et al. [13] for studying cerebral metabolism, have been applied to other tissues and have been suggested to be more reliable in reflecting glucose metabolism than the conventional method of using SUV for quantitation [15, 16]. It is also expected that the maps of the net influx constant of FDG from plasma into tissue (*K*_*i*_) derived from dynamic PET-CT scan provide better contrast between tumor and normal tissues than SUV and hence lead to more accurate tumor segmentation [17]. It has been shown that kinetic analysis using dynamic FDG PET was helpful for diagnosis of central nervous system lymphoma and for differentiation between high-grade glioma and CNS lymphoma [18, 19]. Dimitrakopoulou-Strauss et al. reported that in multiple myeloma, non-small cell lung cancer, and soft-tissue sarcomas the combined use of the parameters by dynamic PET scan and parameters by conventional SUV led to higher accuracy in predicting the treatment outcome [20–22].

We have previously studied the technical feasibility of performing dynamic PET-CT in the evaluation of NPC and found it feasible in characterizing the glucose metabolism of NPC [23]. However, we have not studied the clinical utility of dynamic scan in NPC. The purpose of the study is to evaluate the potential differences in prognostic values of static and dynamic FDG PET-CT scan parameters in NPC patients.

## 2. Material and Methods

### 2.1. Patients

This study was approved by The University of Hong Kong Institutional Review Board. Newly diagnosed patients with histologically proven nonmetastatic NPC were recruited after a written informed consent was obtained. NPC patients were eligible if they met the following criteria: stage M0 according to AJCC staging system [24], serum glucose level < 140 mg/dL before the PET-CT scan, and tumor size > 1 cm in all dimensions to avoid significant partial volume effect.

### 2.2. PET-CT Techniques

All consecutive NPC patients who had conventional whole-body FDG PET-CT scan in our unit and fulfilled the selection criteria between April 2009 and July 2011 were recruited. Patients recruited between October 2010 and July 2011 also had dynamic PET scans. All scans were performed on a hybrid PET-CT scanner (Discovery VCT, GE Healthcare, NJ, USA).

CT scan of the head and neck region including both the carotid arteries and the tumor was first performed using the following protocol: field of view: 50 cm, pixel size: 0.98 × 0.98 × 2.5 mm, spiral CT pitch: 0.984:1, kVp: 120 kV, and gantry rotation speed: 0.5 s, without contrast injection. Then, dynamic PET scan was performed in the same region as the CT scan, starting simultaneously with ^18^F-FDG injection. ^18^F-FDG of 222–370 MBq (5 MBq/kg) was given intravenously. The dynamic PET time sequence comprised 6 frames of 10 seconds each, 4 × 15 seconds, 4 × 30 seconds, 7 × 3 minutes, and 5 × 5 minutes (26 time frames in 50 minutes) in 3D mode with the field of view being 40 cm. Attenuation correction for PET data using CT images was performed and images were reconstructed into 256 × 256 matrix using an ordered-subset expectation maximization iterative algorithm (14 subsets and two iterations). After the dynamic scan and a 10-minute rest, the conventional whole-body PET scan (static PET) was performed with these parameters: 6 bed positions, 2.5 minutes per bed position, 70 cm × 70 cm field of view, and 3D mode.

The same scanning parameters were used for the whole-body PET-CT scan for patients who did not have an additional dynamic PET-CT scan after intravenous administration of ^18^F-FDG (5 MBq/kg) and a 60-minute uptake time.

### 2.3. Image Analysis

According to Patlak et al. [25], *K*_*i*_ is obtained from a plot constructed from the time-activity curves of FDG concentration in plasma and in the tumor, as shown in this equation:

(1) $$\begin{array}{c} \frac{C_{t}\left(t\right)}{C_{p}\left(t\right)}=K_{i}\frac{\int_{0}^{t}C_{p}\left(\tau \right)d\tau }{C_{p}\left(t\right)}+V_{0}, \end{array}$$

where *C*_*t*_(*t*) and *C*_*p*_(*t*) are, respectively, tumor and plasma FDG activity at time *t*; *τ* is the integration time variable; the slope *K*_*i*_ calculated from the regression is the net influx constant; *V*_0_ is a constant representing the initial volume of tracer distribution in both the tissue and blood. The arterial input function *C*_*p*_(*t*) was calculated using the method in our previous publication [23]. For each patient with dynamic scan, the map of *K*_*i*_ was calculated from the dynamic PET images by using the tool imlook4d (free downloaded from http://dicom-port.com/) developed by Jan Axelsson based on Matlab 7 (Mathworks Inc., Natick, MA, USA).

The map of SUV was also calculated from the conventional whole-body PET scan, according to this equation:

(2) $$\begin{array}{c} \text{SUV}=\frac{C\left(t\right)}{D/W_{\text{lbm}}}, \end{array}$$

where *C*(*t*) is the FDG concentration (in MBq/kg or kBq/g) in tissue at time *t* when this last frame of PET scan is performed, *D* is the injected FDG dose (in MBq) at the time of injection (*t* = 0), and *W*_lbm_ is the lean body mass.

The maximal SUV value (SUVmax) and maximal *K*_*i*_ value (*K*_*i*_max) were recorded for each primary NPC lesion. Besides, a series of thresholds, namely, 30%~60% of SUVmax on the static PET images and *K*_*i*_max by dynamic PET scan, were used to delineate the primary NPC lesions from the normal tissues and calculate the tumor volumes as VOL_SUV30_~VOL_SUV60_ and VOL_*K*_*i*_30_~VOL_*K*_*i*_60_, respectively [26, 27].

### 2.4. Treatment and Follow-Up

Three patients had radiotherapy only, and all the other patients had chemoradiotherapy. Radiotherapy was performed with 4~6 MV photon (Varian Medical Systems, Palo Alto, USA). The dose to gross tumor in nasopharynx and involved neck nodes had a range of 68~70 Gy while the dose to the neck had a range of 60~66 Gy in 33~35 fractions. Primary chemotherapy was mainly concurrent cisplatin, 100 mg/sqm at D1, 22, 43 of radiotherapy. Patient may have additional adjuvant or induction chemotherapy for up to 3 cycles with cisplatin (80 or 100 mg/sqm D1) and 5 FU (1000 mg/sqm D1~4 or D1–D5, for adjuvant and induction chemotherapy, resp.). After completion of treatment, patients were followed up every 1-2 months in the first year and every 3–6 months from the second year posttreatment. Nasopharyngeal scope and biopsy were routinely performed at 10 weeks after radiotherapy. The clinical follow-up period was 2.5~51.4 months (mean, 34.1 months; SD, 11.2 months) for this NPC cohort. The patients who died or had residual disease or recurrence during the follow-up period were considered to have “poor outcome,” and those without evidence of disease on routine nasopharyngeal biopsy and on subsequent clinical follow-up were considered to have “good outcome.”

### 2.5. Statistical Analysis

The correlation between SUVmax and *K*_*i*_max was evaluated by using Pearson's correlation. The difference between SUV-derived tumor volumes (VOL_SUV_) and *K*_*i*_-derived tumor volumes (VOL_*K*_*i*__) was analyzed by using two-sample *t*-test and Bland-Altman analysis.

To study the prognostic value of the PET-CT parameters, that is, SUVmax, *K*_*i*_max, and various metabolic tumor volumes (VOL_SUV30_~VOL_SUV60_ and VOL_*K*_*i*_30_~VOL_*K*_*i*_60_), the cut-off value of each parameter was determined by receiver operating characteristics (ROC) analysis and all patients were then divided into two groups by this cutoff. Fisher's exact test was employed to compare the statistical proportions of poor outcome or good outcome in these two subgroups. Furthermore, multivariate ROC analysis was performed to evaluate the value of different factors or different combinations of factors in predicting the treatment outcome. The binary logistic regression algorithm was used for different combinations, and the predicted probability was produced and used as a new variable. Finally this new variable was reentered as the test variable in the ROC analysis [28].

All statistical analyses (except ROC curve comparison) were performed using PASW Statistics 20 (IBM Corporation, Armonk, NY). The comparison of ROC curve was performed in MedCalc (MedCalc Software, Mariakerke, Belgium). A two-sided *p* value less than 0.05 was considered statistically significant.

## 3. Results

Forty-five NPC patients, including 29 with static whole-body PET-CT scan only and 16 patients with both the static and dynamic PET-CT scan, were recruited. Patient demographics and clinical details are shown in Table 1. The mean age of this cohort was 52.5 years (SD 12.3 years, range 28.0~79.0 years). SUVmax ranged from 3.6 to 24.0 (mean, 9.7; SD, 5.1), and VOL_SUV40_ ranged from 2.4 to 60.1 cm^3^ (mean, 15.2; SD, 11.3).

In the 45 cases cohort, 8 had poor outcome. By testing a series of thresholds (30%~60% of SUVmax), it was found that VOL_SUV40_ was a significant predictor of treatment outcome according to Fisher's exact test; with a cutoff of 20 cm^3^, the large tumor volume group (VOL_SUV40_ > 20 cm^3^) had a significantly higher proportion of patients with poor outcome than the small tumor volume group (50%* versus* 11%, *p* = 0.024). SUVmax was not significant in Fisher's exact test.

**Figure 1:**
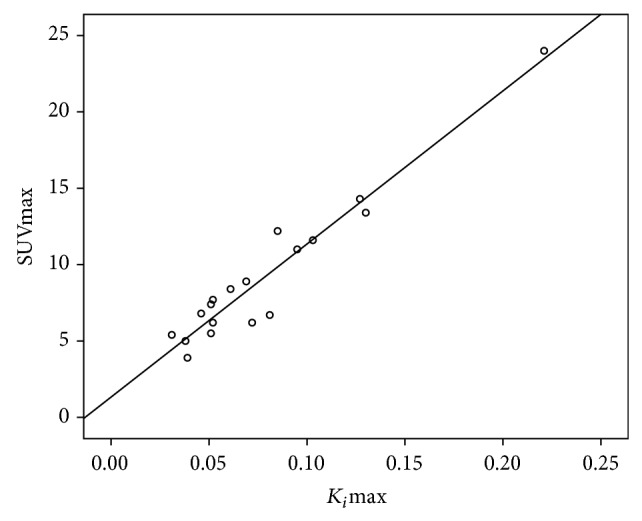
Scatter plots show significant relationship by Pearson's correlation between SUVmax and *K*_*i*_max with *r* = 0.964, *p* < 0.001.

The details of patients with dynamic PET-CT scan are shown in Table 2. There are 12 males and 4 females with a median age of 50.0 years and a range of 31.0~69.0 years. Of the 16 patients, two had residual disease whilst 14 were alive without evidence of disease at last follow-up. SUVmax ranged from 3.9 to 24.0 (mean, 8.8; SD, 5.0); *K*_*i*_max ranged from 0.031 to 0.221 min^−1^ (mean, 0.076 min^−1^; SD, 0.049 min^−1^); VOL_SUV40_ ranged from 5.2 to 27.5 cm^3^ (mean, 16.0 cm^3^; SD, 6.8 cm^3^). SUVmax and *K*_*i*_max were highly correlated (*r* = 0.970, *p* < 0.001) as shown in Figure 1. None of the parameters from dynamic scan was significant in predicting the treatment outcome according to Fisher's exact test (for 16 patients with dynamic scan only).

**Figure 2:**
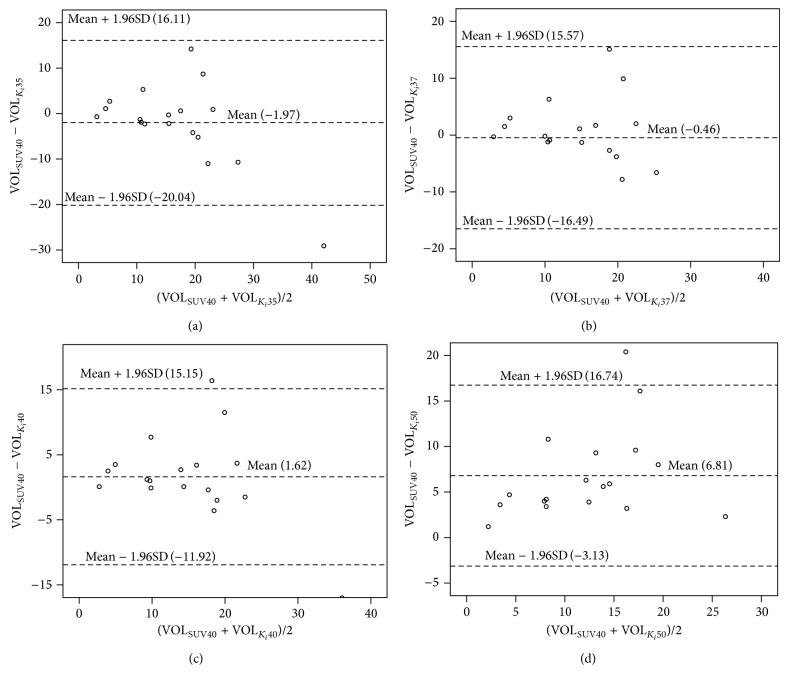
Agreement between static PET-CT and dynamic PET-CT with different thresholds in the estimation of primary tumor volumes; (a) PET volume with the threshold of 40% SUVmax (VOL_SUV40_) versus PET volume with the threshold of 35% *K*_*i*_max (VOL_*K*_*i*_35_), (b) VOL_SUV40_ versus PET volume with the threshold of 37% *K*_*i*_max (VOL_*K*_*i*_37_), (c) VOL_SUV40_ versus PET volume with the threshold of 40% *K*_*i*_max (VOL_*K*_*i*_40_), and (d) VOL_SUV40_ versus PET volume with the threshold of 50% *K*_*i*_max (VOL_*K*_*i*_50_). The mean of the two volumes compared is plotted against the difference, and the middle line in the figures is the mean of the differences, whereas the upper and lower lines represent the 95% confidence intervals (mean ± 1.96 × SD).

By testing a series of thresholds (30%~60% of *K*_*i*_max), it was found that with the threshold of 37% *K*_*i*_max, the tumor volume (VOL_*K*_*i*_37_) was not significantly different from VOL_SUV40_ (*p* = 0.773, paired *t*-test). VOL_*K*_*i*_37_ ranged from 3.7 to 51.6 cm^3^ (mean, 16.7 cm^3^; SD, 11.6 cm^3^).  Figure 2 shows the results of Bland-Altman analysis of the difference between the tumor volumes with a series of thresholds. Tumor volumes measured using VOL_*K*_*i*_37_ were closest to tumor volumes measured using VOL_SUV40_ and the differences were within the confidence interval (Figure 2(b)). As shown in Table 2, tumor volumes by *K*_*i*_ maps were correlated with VOL_SUV40_ by SUV maps with Pearson's correlation coefficient of 0.684, 0.689, 0.699, and 0.688 for VOL_*K*_*i*_35_, VOL_*K*_*i*_37_, VOL_*K*_*i*_40_, and VOL_*K*_*i*_50_, respectively. With the same threshold of 40% on both *K*_*i*_ maps and SUV maps, it was observed that VOL_*K*_*i*_40_ has a tendency to be bigger than VOL_SUV40_ (Figure 2(c)).

Table 3 shows the results of multivariate ROC analysis for evaluating the static PET-CT (SUVmax, VOL_SUV40_) and dynamic PET-CT parameters (*K*_*i*_max, VOL_*K*_*i*_37_) in classifying the patients with poor outcome or good outcome. VOL_SUV40_ achieved the highest predicting accuracy (88%) among these four parameters. The combination of SUVmax with VOL_SUV40_ did not increase the predicting value, while the combination of VOL_SUV40_ and VOL_*K*_*i*_37_ showed a tendency of higher AUC (area under curve in ROC analysis); however, the difference between the AUCs of the two ROC curves was insignificant (*p* = 0.832). The combination of all these four parameters showed the highest accuracy (100%* versus* 88%) and the highest area under the ROC curve (1.000* versus* 0.893) compared to VOL_SUV40_ alone, indicating that this combination may predict better in treatment outcome. However, the difference between the AUCs of the two ROC curves was also found insignificant (*p* = 0.768).

## 4. Discussion

Our results showed that in this NPC cohort, the net influx constant *K*_*i*_max from Patlak graphical analysis was highly correlated with SUVmax in the primary tumor. This correlation echoes previous publications and has been explained well in the literature [29–32]. Briefly, according to the kinetic model [25], *K*_*i*_ stands for the net flux of FDG that is transported from plasma into the tissue and then metabolized, while SUV is the overall FDG uptake in a voxel or a region normalized to injected dose and body weight [10]. The significant correlation indicates that the overall FDG uptake is mainly composed of the metabolized FDG in tissue. However, there are some differences between SUVmax and *K*_*i*_. As shown in the results of the correlation analysis in our cohort, 94% (*R* ^2^ = 0.97∗0.97 = 0.94) of the SUVmax was determined by *K*_*i*_ and 6% was not. This can be explained by the following reasons: *K*_*i*_ reflects only the uptake rate of FDG which is metabolized while SUV measurement also includes the unmetabolized fraction of FDG; *K*_*i*_ accounts for the FDG available to the tumor cells while SUV accounts for the overall FDG uptake including the FDG in plasma [30].

In our study, we found that with the threshold of 40% *K*_*i*_max, the metabolic volumes were systematically bigger than the volumes measured using a threshold of 40% SUVmax (Figure 2). This finding was in accordance with the previous results comparing these parameters in lung cancer and gastrointestinal cancer. In lung cancer, it was reported that for all lesions, with the same threshold of 50%, the volumes by the maps of glucose metabolism rate (this parameter is calculated by multiplying *K*_*i*_ by a constant for the subject) were significantly smaller than the volumes by SUV maps [17]. In another paper comparing the metabolic tumor volumes obtained from static PET and dynamic PET images, generally the SUV-derived tumor volumes were bigger than the *K*_*i*_-derived tumor volumes [33]. It was proposed that there might be lower background intensity in *K*_*i*_ maps [34]. One reason for the higher background intensity in SUV images is that SUV uptake consists not only of trapped or metabolized FDG but also of free and nonmetabolized FDG which exists in blood vessels or the intercellular space and so forth. This finding is noteworthy when using the *K*_*i*_ maps for the calculation of metabolic tumor volumes. This comparison between the volumes derived by *K*_*i*_ map or SUV map also indicates that VOL_*K*_*i*__ may have more potential in radiotherapy planning because with *K*_*i*_ map the boundary can be more clear-cut and so the delineation can be more accurate [34].

PET-CT derived metabolic tumor volume may have an impact on the management of patients with NPC in terms of prognostication. It was reported that PET-derived primary tumor volumes by a threshold of 50% SUVmax were useful in predicting the patient outcomes with larger metabolic tumor volumes associated with shorter overall survival [26]. Recently, the volume by a SUV value of 2.5 was also shown to be an independent risk factor in predicting the PFS and OS in metastatic NPC patients by Chan et al. [35]. Our result was generally in accordance with these reported findings and has shown the predicting value of metabolic tumor volume given the appropriate threshold of the metabolic PET parameter in the prognosis of NPC patients.

To analyze the role of dynamic scan in improving the predictive value and as an adjunct parameter, multivariate ROC analysis based on binary regression was performed to study the impact of the combination of these factors. Although the combination of all four parameters, and the combination of only VOL_SUV40_ and VOL_*K*_*i*_37_, tended to achieve higher accuracy than VOL_SUV40_ only, a significant difference was not achieved. In some cancer types, this combination of parameters produces higher predicting value in patient survival [20, 21, 36]. Dimitrakopoulou-Strauss et al. reported that in patients with multiple myeloma the combined use of several predictor variables, namely, SUV, *k*_3_, and fractal dimension (the last two were from dynamic scan) led to the highest correct classification rate of the group with longer survival [20], and in the patients with metastatic soft-tissue sarcomas the combined use of mean SUV and *K*_1_ led to the highest accuracy of the classification between the nonresponders and responders [21]. In our study, the improvement, albeit small, suggests that the combination of dynamic and static PET parameters may improve the accuracy of prognostication in NPC patients. It is possible that the insignificant findings may be due to the statistical error caused by the small cohort of 16 patients and the much smaller group of only 2 patients with poor outcome. We hence concluded that the usefulness of dynamic scan was not proven in our study. Larger studies with longer follow-up are suggested.

## 5. Conclusion

In summary, the results of this study showed that the glucose metabolism parameters (*K*_*i*_ versus SUV; VOL_*K*_*i*__ versus VOL_SUV_) by dynamic and static scanning of PET-CT were significantly correlated. Metabolic tumor volume using a threshold of SUVmax 40% (VOL_SUV40_) from static scan is useful in predicting patient outcome. However the role of dynamic PET-CT scanning in predicting outcome was not justified in this small NPC cohort.

## Figures and Tables

**Table 1:** Patient characteristics (*n* = 45; 16 patients with dynamic scan included).

Baseline characteristic	
Age (years)	
Range	28~79
Median	52.5
Standard deviation	12.3
Gender	
Number of females	11 (24%)
Number of males	34 (76%)

Stage information	Number of patients (%)

T-stage	
1	4 (9%)
2	7 (15%)
3	25 (56%)
4	9 (20%)
N-stage	
0	1 (2%)
1	6 (14%)
2	27 (60%)
3	11 (24%)

**Table 2:** Details of the NPC cohort with dynamic PET-CT (*n* = 16).

Number	Age	Sex	SUVmax	*K*_*i*_max	VOL_SUV40_	VOL_*K*_*i*_37_	*T*	*N*	Status
1	39	M	6.2	0.052	25.7	15.8	3	3	NED
2	31	F	7.7	0.052	27.5	51.6	4	3	NED
3	38	M	13.4	0.130	14.4	15.7	2b	1	NED
4	51	M	3.9	0.039	13.7	7.4	1	2	NED
5	55	M	11.0	0.095	10.2	11.1	3	3	NED
6	34	F	6.2	0.072	9.8	11.0	4	2	NED
7	42	M	7.4	0.051	17.9	21.7	3	1	NED
8	64	M	8.9	0.069	17.5	20.2	2	2	NED
9	65	M	5.4	0.031	17.8	16.1	3	2	NED
10	69	M	6.8	0.046	16.7	24.5	1	2	NED
11	43	M	24.0	0.221	9.9	10.1	4	2	NED
12	38	F	5.5	0.051	22.0	28.6	3	3	AWD
13	61	M	14.3	0.127	6.7	3.7	3	2	NED
14	57	M	8.4	0.061	15.3	14.2	3	3	NED
15	63	M	5.0	0.038	26.4	11.3	3	3	AWD
16	50	F	6.7	0.081	5.2	3.7	3	2	NED

Notes: age (years), age at diagnosis; SUVmax, the maximum standardized uptake value; *K*_*i*_max (min^−1^), the maximum net influx constant of FDG from plasma into tissue; VOL_SUV40_, tumor volume in cm^3^ calculated in SUV maps using the threshold of 40% SUVmax; VOL_*K*_*i*_37_, tumor volume in cm^3^ calculated in *K*_*i*_ maps using the threshold of 37% *K*_*i*_max; TNM, tumor-node metastases stage by AJCC staging system; NED = no evidence of disease; AWD = alive with disease.

**Table 3:** Results of receiver operating characteristics (ROC) analysis for studying the static PET-CT and dynamic PET-CT parameters in classifying the patients (*n* = 16) with poor outcome and good outcome.

Parameter	Sen	Spe	Acc	AUC
SUVmax	100%	79%	81%	0.857
VOL_SUV40_	100%	86%	88%	0.893
SUVmax & VOL_SUV40_	100%	86%	88%	0.893
*K*_*i*_max	100%	71%	75%	0.839
VOL_*K*_*i*_37_	100%	43%	50%	0.679
*K*_*i*_max & VOL_*K*_*i*_37_	100%	71%	75%	0.821
SUVmax & *K*_*i*_max	100%	79%	81%	0.857
VOL_SUV40_ & VOL_*K*_*i*_37_	100%	86%	88%	0.929
SUVmax & *K*_*i*_max & VOL_SUV40_ & VOL_*K*_*i*_37_	100%	100%	100%	1.000

Notes: Sen, sensitivity; Spe, specificity; Acc, accuracy; AUC, area under the ROC curve.
